# Cerebrospinal fluid electrolytes and acid-base in diabetic patients

**DOI:** 10.1515/tnsci-2020-0196

**Published:** 2021-11-09

**Authors:** Chia-Chih Liao, Te-Hsin Hou, Huang-Ping Yu, Allen Li, Fu-Chao Liu

**Affiliations:** Department of Anesthesiology, Chang Gung Memorial Hospital, No. 5, Fushing 1st Rd, Gueishan, Taoyuan 33305, Taiwan; College of Medicine, Chang Gung University, Taoyuan, Taiwan

**Keywords:** cerebrospinal fluid, electrolyte, acid-base, diabetes mellitus

## Abstract

**Background:**

Diabetes mellitus (DM) has detrimental effects on the function of microvascular beds, resulting in blood–brain barrier (BBB) dysfunction. The objective of the study was to investigate whether DM affects the brain physiology through composition of cerebrospinal fluid (CSF) and compare gas tension and electrolyte levels in CSF between the diabetic and nondiabetic populations.

**Methods:**

Patients aged between 20 and 70 years scheduled for elective orthopedic or urologic surgery requiring spinal anesthesia were enrolled. They were assigned to either of the two groups (control or type 2 DM). Gas tension and electrolytes in the CSF and whole blood samples were measured in both groups.

**Results:**

All 49 enrolled patients (24 in the control and 25 in the DM group) completed the study. The concentrations of Na^+^ and Mg^2+^ in the blood were significantly lower in the DM group than those in the control. The levels of pCO_2_ and {\text{HCO}}_{3}^{-}] in the CSF were lower in the DM group than in the control group. In addition, there was a marked increase in the glucose level in both the blood and CSF in the DM group.

**Conclusion:**

The results show that there were some homeostatic changes in blood and CSF in patients with DM.

## 1 Introduction

Cerebrospinal fluid (CSF) fills the spaces between the brain and spinal cord, flowing through the brain ventricles and subarachnoid space. It is a major component of the extracellular fluid in the central nervous system (CNS). Due to its special location and interaction with the brain parenchyma, CSF has many important roles in brain function. An analysis of CSF composition may assist clinicians identify patients with CNS disorders [1,2].

Diabetes mellitus (DM) is a major public health issue worldwide. It is a metabolic disorder that results from defects in insulin production (type 1, insulin-dependent) and insulin resistance (type 2, insulin-independent). DM has detrimental effects on the function of vascular beds, resulting in various peripheral and CNS complications. Recent evidence from clinical and experimental studies suggested that prolonged hyperglycemic status induced tissue damage and elicited a progressive injury of the brain through several different pathogenetic mechanisms [3,4]. It has been reported that hyperglycemia and abnormal glucose metabolism lead to the generation of reactive oxygen species and elicit inflammatory responses, resulting in endothelial injury of the cerebral microvasculature [5,6]. Altered glycemic conditions in diabetic patients may contribute to the early breakdown of the blood–brain barrier (BBB) [7]. The BBB is a highly specialized endothelial structure in the brain that helps maintain the brain homeostasis by separating the circulating blood components from neurons. Recent studies have shown that patients with diabetes have related changes in BBB integrity. In animal models, hyperglycemia significantly exacerbates BBB dysfunction and edema formation after focal ischemia-reperfusion injury in the brain [8]. MRI images of the brain in DM patients also showed increased BBB permeability when compared to controls [9].

CSF is mainly produced by the choroid plexus via active transport instead of passive ultrafiltration [10]. It regulates the distribution of substances or metabolic waste products in different areas of the brain. Although the respective compositions in blood and CSF are quite similar, there does exist a small difference between them. Breakdown of the BBB alters enzymatic activity and transport of molecules between the blood and brain, leading to neuronal dysfunction and loss, as seen in many CNS disorders such as Alzheimer’s disease [11] and traumatic brain injury [12]. DM is characterized by neurovascular dysfunction involving an inflammatory cascade and BBB breakdown. Clinical manifestations in patients with DM include cognitive, learning, memory deficits, and structural brain abnormalities [13,14]. To our knowledge, there is limited information about ion homeostasis in the CNS, which may be crucial in the pathophysiology of brain dysfunction in DM patients. Therefore, the objective of the present study was to investigate how DM affects the brain physiology through CSF composition. In addition, the gas tension and electrolyte parameters in CSF between the DM and non-DM populations were compared.

## 2 Materials and methods

### 2.1 Patients

Forty-nine patients with American Society of Anesthesiologists physical status I or II scheduled for elective urologic or orthopedic surgery and receiving spinal anesthesia were enrolled in this controlled study. Of the 49 patients, while 25 had a history of type 2 DM and were taking oral hypoglycemic agents, the other 24 (control group) had no history of type 2 DM. All patients were between 20 and 70 years. Patients with contraindications for spinal anesthesia, severe cardiovascular dysfunction, renal dysfunction, type 1 DM, any history of neuropathy or neurodegenerative diseases, CNS infection, sepsis, or known allergies to any medications used in this study were excluded.


**Informed consent:** Informed consent has been obtained from all individuals included in this study.
**Ethical approval:** The research related to human use has been complied with all the relevant national regulations, institutional policies, and in accordance with the tenets of the Helsinki Declaration and has been approved by the Institutional Review Board of Chang Gung Memorial Hospital, Taoyuan, Taiwan (IRB: 1602240003).

### 2.2 Study protocol

According to DM status, the patients were assigned to the two groups (control or type 2 DM). All fasted for at least 8 h before sampling blood and CSF. In the operating room, standard monitoring, including noninvasive arterial blood pressure, electrocardiography, and pulse oximetry, was applied. The patient was positioned in the lateral decubitus position. Spinal anesthesia was performed at the L3–4 or L4–5 interspace with a 25-gauge Quincke-type needle through the midline. After discarding an initial volume of approximately 1 mL, another 1 mL of CSF was sampled, following which 15 mg of 0.5% isobaric bupivacaine was administered by an experienced anesthesiologist. After the regional anesthesia was complete, the patient was restored in the supine position. A whole blood sample (2.5 mL) was also collected through arterial puncture from the femoral or dorsalis pedis artery before the operation. CSF and whole blood samples were collected using commercial blood gas syringes with dry heparin preparation. Next, blood gas and electrolyte analyses of CSF and whole blood samples were performed using a StatProfile^®^ Critical Care Xpress Analyzer (Nova Biomedical, Waltham, MA, USA).

### 2.3 Statistical analysis

All data analyses were performed using SPSS version 17.0 (SPSS Statistics for Windows; SPSS Inc., Chicago, IL). Data were assessed for normality using the Kolmogorov-Smirnov test. Categorical data were reported as numbers and percentages and analyzed using the chi-square test with the Yates correction. Continuous data were expressed as the mean ± standard deviation. While the parametric data were analyzed using an independent *t*-test, the Mann–Whitney *U*-test was employed for nonparametric data. Multivariate logistic regression was applied with adjustment for confounding factors, including age, sex, weight, and height. Differences were considered statistically significant at *p* < 0.05.

## 3 Results

**Figure 1:**
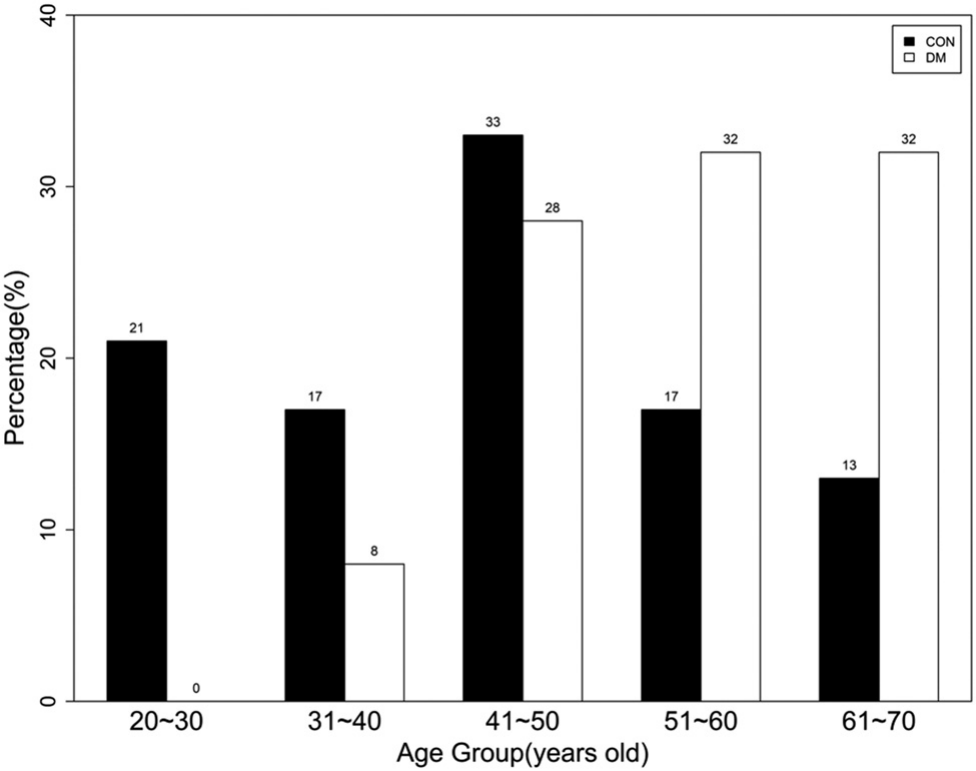
Percentiles in different age categories in control and DM groups.

All 49 enrolled patients (24 in the control and 25 in the DM group) scheduled for elective urologic or orthopedic surgery and receiving spinal anesthesia completed the study. CSF and whole blood samples were collected from all the patients. The percentiles for the age categories are shown in Figure 1. In the control group, more samples were collected from patients aged 41 to 50 years than from the other age groups. In the DM group, most samples were collected from those between 41 and 70 years, and over 50% were above 51 years.

### 3.1 Patient characteristics in the control and DM groups

The characteristics of patients included in the two groups are depicted in Table 1. As seen from the table, the age in the DM group was higher than that in the control group (54.7 ± 10.0 vs 44.7 ± 14.0 years, *p* = 0.006). As compared to the control group, there were more males in the DM group (*p* = 0.001). In the DM group, weight and height of patients were higher as compared with those in the control group (71.4 ± 13.6 vs 61.7 ± 10.9 kg, *p* = 0.008; 168.5 ± 8.2 vs 163.0 ± 6.2 cm, *p* = 0.011, respectively). There was no significant difference in the preoperative blood pressure, heart rate, and oxyhemoglobin saturation between the two groups.

**Table 1:** Patient characteristics

	Control group (*n* = 24)	DM group (*n* = 25)	*p* value
Age (years)	44.7 ± 14.0	54.7 ± 10.0	0.006^*^
M/F	9(37.5)/15(62.5)	21(84.0)/4(16.0)	0.001^*^
Weight (kg)	61.7 ± 10.9	71.4 ± 13.6	0.008^*^
Height (cm)	163.0 ± 6.2	168.5 ± 8.2	0.011^*^
BMI (kg/m^2^)	23.2 ± 3.6	25.2 ± 4.8	0.107
Preoperative			
SBP (mmHg)	119.3 ± 19.1	123.3 ± 23.2	0.349
DBP (mmHg)	71.1 ± 11.1	71.4 ± 12.7	0.504
HR (min)	70.5 ± 10.4	68.5 ± 10.2	0.911
SpO_2_ (%)	99.46 ± 0.59	99.52 ± 0.59	0.983

M = male; F = female; SBP: systolic blood pressure; DBP: diastolic blood pressure; HR: heart rate; SpO_2_: oxyhemoglobin saturation by pulse oximetry.

Data are reported as mean ± standard deviation or number (%).

*p < 0.05 when compared with the control group.

### 3.2 Comparison of gas tension, electrolyte variables, and glucose in blood between the control and DM groups

The results of the gas tension analysis, electrolyte variables, and blood glucose levels in the two groups are presented in Table 2. There was no significant difference in gas tension between the two groups. As compared to the control group, the concentration of Na^+^ and Mg^2+^ in blood was significantly lower in the DM group (133.71 ± 3.49 vs 136.74 ± 2.98 mEq/L, *p* = 0.005; 0.45 ± 0.10 vs 0.56 ± 0.06 mEq/L, *p* = 0.045). The glucose level was markedly increased in the DM group compared with that in the control group (171.60 ± 62.56 vs 123.33 ± 44.08 mg/dL, *p* = 0.041). Additionally, no significant differences in the levels of K^+^, Cl^−^, Ca^2+^, and lactate were observed between the two groups.

**Table 2:** Comparison of gas tension, electrolyte variables, and glucose in blood between control and DM groups

	Control group (*n* = 24)	DM group (*n* = 25)	Adjusted *p* value
pH	7.40 ± 0.03 (7.39, 7.42)	7.42 ± 0.04 (7.41, 7.44)	0.556
pCO_2_ (mmHg)	35.44 ± 4.93 (33.36, 37.52)	33.81 ± 5.61 (31.49, 36.412)	0.984
pO_2_ (mmHg)	131.13 ± 67.12 (102.8, 159.53)	110.54 ± 77.71 (78.46, 142.61)	0.317
{\text{HCO}}_{3}^{-}] (mEq/L)	22.24 ± 2.06 (21.37, 23.11)	22.14 ± 3.19 (20.82, 23.46)	0.680
BE (mEq/L)	−1.44 ± 1.46 (−2.06, −0.82)	−1.26 ± 2.86 (−2.44, −0.08)	0.637
BUN (mg/dL)	11.92 ± 3.87 (10.28, 13.55)	20.08 ± 16.42 (13.30, 26.86)	0.325
Na^+^ (mEq/L)	136.74 ± 2.98 (135.51, 138.04)	133.71 ± 3.49 (132.31, 135.24)	0.005^*^
K^+^ (mEq/L)	3.68 ± 0.33 (3.54, 3.82)	3.92 ± 0.30 (3.79, 4.05)	0.055
Cl^−^ (mEq/L)	109.43 ± 2.35 (108.41, 110.44)	108.62 ± 4.33 (106.81, 111.44)	0.218
Ca^2+^ (mEq/L)	1.15 ± 0.05 (1.13, 1.17)	1.17 ± 0.07 (1.14, 1.20)	0.133
Mg^2+^ (mEq/L)	0.56 ± 0.06 (0.51, 0.62)	0.45 ± 0.10 (0.40, 0.50)	0.045^*^
Glucose (mg/dL)	123.33 ± 44.08 (104.71, 141.94)	171.60 ± 62.56 (145.81, 197.44)	0.041^*^
Lac (mmol/L)	1.16 ± 0.52 (0.86, 1.46)	1.02 ± 0.48 (0.78, 1.26)	0.479

BE: base excess; Lac: lactate.

Data are reported as mean ± standard deviation (95% CI).

*p* value is adjusted for age, gender, weight, and height.

*p < 0.05 when compared with the control group.

### 3.3 Comparison of gas tension, electrolyte variables, and glucose in lumbar CSF between the control and DM groups

The values of gas tension analysis, electrolyte variables, and glucose in the lumbar CSF from the two groups are shown in Table 3. There was no significant difference in the pH of the CSF between the two groups, and it was slightly alkaline in both groups. The levels of pCO_2_ and {\text{HCO}}_{3}^{-}] were lower in the DM group as compared with the control group (31.41 ± 3.77 vs 34.61 ± 4.90 mmHg, *p* = 0.012; 21.24 ± 2.37 vs 23.48 ± 2.32 mEq/L, *p* = 0.023, respectively). In both groups, the higher pH of the CSF is attributed to its lower pCO_2_, reflecting a decreased bicarbonate buffering capacity. As regard the electrolyte values of CSF, there was no significant difference in the levels of Na^+^, K^+^, Cl^−^, Ca^2+^, Mg^2+^, and lactate between the two groups. As expected, the glucose level was significantly higher in the DM group than that in the control group (83.20 ± 23.75 vs 55.04 ± 5.90 mg/dL, *p* = 0.013).

**Table 3:** Comparison of gas tension, electrolyte variables, and glucose in lumbar CSF between control and DM groups

	Control group (*n* = 24)	DM group (*n* = 25)	Adjusted *p* value
pH	7.43 ± 0.02 (7.42, 7.45)	7.44 ± 0.03 (7.43, 7.44)	0.415
pCO_2_ (mmHg)	34.61 ± 4.90 (32.54, 36.68)	31.41 ± 3.77 (29.85, 32.97)	0.012^*^
pO_2_ (mmHg)	122.61 ± 23.46 (112.74, 132.52)	126.35 ± 16.91 (119.39, 133.32)	0.165
{\text{HCO}}_{3}^{-}] (mEq/L)	23.48 ± 2.32 (7.39, 7.42)	21.24 ± 2.37 (7.39, 7.42)	0.023^*^
BE (mEq/L)	0.35 ± 1.60 (−0.32, 1.03)	−1.58 ± 1.95 (−2.41, −0.76)	0.014^*^
BUN	10.96 ± 2.40 (10.14, 12.13)	16.64 ± 12.23 (11.59, 21.69)	0.262
Na^+^ (mEq/L)	137.17 ± 1.81 (136.39, 137.49)	136.20 ± 2.89 (135.09, 137.42)	0.331
K^+^ (mEq/L)	2.75 ± 0.08 (2.71, 2.78)	2.80 ± 0.13 (2.75, 2.86)	0.485
Cl^−^ (mEq/L)	121.79 ± 1.86 (121.03, 122.62)	120.84 ± 2.27 (119.89, 121.82)	0.379
Ca^2+^ (mEq/L)	0.98 ± 0.03 (0.97, 0.99)	0.99 ± 0.04(0.98, 1.01)	0.422
Mg^2+^ (mEq/L)	0.78 ± 0.03 (0.76, 0.79)	0.76 ± 0.04 (0.74, 0.78)	0.971
Glucose (mg/dL)	55.04 ± 5.90 (52.55, 57.53)	83.20 ± 23.75 (73.39, 93.00)	0.013^*^
Lac (mmol/L)	1.12 ± 0.13 (1.05, 1.19)	1.31 ± 0.27 (1.18, 1.43)	0.598

BE: base excess; Lac: lactate.

Data are reported as mean ± standard deviation (95% CI).

*p* value is adjusted for age, gender, weight, and height.

*p < 0.05 when compared with the control group.

### 3.4 Comparison of age subgroups in DM patients

In the DM group, nine of 25 patients were between 31 and 50 years, and the age of the remaining 16 ranged from 51 to 70 years (36 and 64%, respectively). We compared the gas tension and biochemical variables in the blood and lumbar CSF in the two age subgroups (Table 4). It was seen that there were no significant differences between the two age subgroups.

**Table 4:** Comparison of gas tension, electrolyte variables, and glucose in blood and lumbar CSF between age subgroups in DM patients

Age subgroup	Blood			Lumbar CSF		
	Age <51 years (*n* = 9)	Age ≥51 years (*n* = 16)	*p* value	Age <51 years (*n* = 9)	Age ≥51 years (*n* = 16)	*p* value
pH	7.42 ± 0.04	7.42 ± 0.04	0.597	7.43 ± 0.02	7.44 ± 0.02	0.849
pCO_2_ (mmHg)	34.03 ± 5.05	33.68 ± 6.06	0.884	31.32 ± 3.16	31.46 ± 4.18	0.931
pO_2_ (mmHg)	121.48 ± 63.18	126.88 ± 62.11	0.365	119.89 ± 12.96	129.98 ± 18.14	0.156
{\text{HCO}}_{3}^{-}] (mEq/L)	21.97 ± 2.54	22.24 ± 3.58	0.843	21.09 ± 1.60	21.32 ± 2.75	0.821
BE (mEq/L)	−1.52 ± 2.32	−1.11 ± 3.18	0.739	−1.64 ± 1.22	−1.55 ± 2.33	0.909
BUN (mg/dL)	21.89 ± 16.43	19.06 ± 16.86	0.689	18.78 ± 13.30	15.44 ± 11.87	0.524
Na^+^ (mEq/L)	134.10 ± 1.63	133.49 ± 4.23	0.615	135.94 ± 3.70	136.33 ± 2.52	0.761
K^+^ (mEq/L)	3.97 ± 0.34	3.89 ± 0.29	0.534	2.81 ± 0.10	2.80 ± 0.14	0.758
Cl^−^ (mEq/L)	108.30 ± 3.41	108.81 ± 4.86	0.785	120.59 ± 2.78	120.99 ± 2.03	0.683
Ca^2+^ (mEq/L)	1.18 ± 0.11	1.17 ± 0.05	0.656	0.99 ± 0.05	1.00 ± 0.03	0.497
Mg^2+^ (mEq/L)	0.44 ± 0.09	0.49 ± 0.11	0.382	0.76 ± 0.05	0.77 ± 0.04	0.775
Glucose (mg/dL)	172.11 ± 68.77	155.56 ± 54.57	0.514	85.11 ± 26.68	82.13 ± 22.79	0.770
Lac (mmol/L)	1.03 ± 0.57	1.01 ± 0.41	0.926	1.29 ± 0.30	1.32 ± 0.24	0.807

BE: base excess; Lac: lactate.

Data are reported as mean ± standard deviation.

### 3.5 Relation between Na^+^ CSF/blood ratio and blood glucose levels

**Figure 2:**
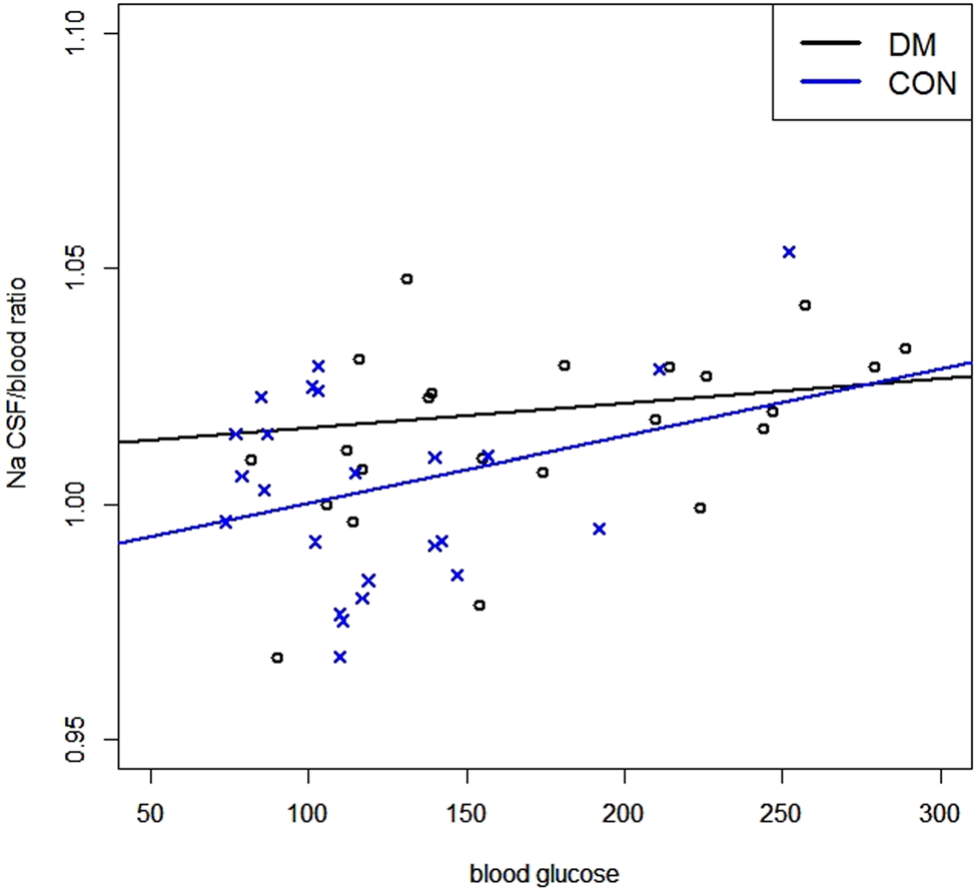
The linear correlation between the values of Na+ CSF/blood ratio and blood glucose in control and DM groups.

A linear correlation between the Na^+^ CSF/blood ratio and blood glucose is depicted in Figure 2. The ratio increased with blood glucose in both control and DM groups (*r* = 0.299 and 0.113, respectively; *p* < 0.05).

## 4 Discussion

In this study, CSF samples for profile analysis of patients requiring spinal anesthesia were collected. We analyzed the gas tension and electrolyte parameters of blood and CSF in DM and non-DM adult patients, and some differences between the two groups were seen. It was seen that the concentration of Na^+^ and Mg^2+^ was lower in the blood of the DM group than that in the control group. In addition, there was a marked increase in the glucose level in both the blood and CSF in the DM group.

Diabetes is one of the largest epidemics worldwide. Globally, the number of people with type 2 DM has doubled in the past 20 years. With respect to age, a recent meta-analysis study found that the prevalence rate of type 2 diabetes was seven times higher in the 55–74 age group than that in the 20–34 group for Chinese adults, and similar prevalence rates were observed in both women and men [15]. In our prospective controlled study, the patients in the DM group were older than those in the control group (*p* = 0.006); approximately 60% of the patients were above 51 years, which is in line with the age distribution pattern in a previous meta-analysis study. Moreover, there were more males than females in the DM group. This may be because more patients scheduled for the urologic surgery and enrolled in the study were males. Therefore, for comparison, we adjusted the data for age and sex. Furthermore, in another previous study, the values of serum Na^+^ and K^+^ revealed a significant positive correlation with the age of patients [16]. However, in our study no significant difference in study variables was observed between the two age subgroups. This difference might be due to the renal dysfunction status; almost 50% patients had renal dysfunction in the previous study and those enrolled in our study had normal renal function.

Maintaining the pH of the CSF in a narrow range near 7.4 is important for homeostasis because the neurons in the CNS are highly susceptible to small changes in acid-base balance [17]. Many conditions, including respiratory acidosis/alkalosis or metabolic events, may lead to variations in CSF. In patients with both type 1 and type 2 diabetes, the incidence of lactic acidosis is reported to be 3% [18]. Diabetic ketoacidosis is also a severe complication observed in the diabetic population. The variations in pH of the CSF in response to changes in blood pH are related to the function of the BBB. The BBB is relatively freely permeable to CO_2_, but not to hydrogen or {\text{HCO}}_{3}^{-}] ions. Rapid compensation occurs through the central chemoreceptors in the medulla oblongata, which influence respiratory activity by changing the pCO_2_ levels. The central canal of the spinal cord also has lining neurons that are sensitive to both acidic and alkaline pH, which intrinsically regulates the pH of the CSF [19]. In our study, no significant difference (*p* = 0.415) in the CSF pH was observed between the two groups, although both were slightly alkaline. The higher pH in CSF contributes to its lower pCO_2_ and compensates for the decreased buffering capacity of the bicarbonate.

CSF is mainly produced by the choroid plexus. The respective distributions of main ions between blood and CSF are similar as seen in our study. We also observed that the respective concentrations of Na^+^ were very close in the blood and CSF in the control group, but not in the DM group. Likewise, the concentrations of K^+^ and Ca^2+^ were higher in the blood, whereas the concentrations of Cl^−^ and Mg^2+^ were lower than those in the CSF.

The secretion of CSF across the choroid plexus occurs through active unidirectional flux via transporters and ion channels and not by the passive ultrafiltration of the plasma [10]. This may have been the reason for the concentrations of ions in the CSF being virtually constant, despite variations in the concentration of ions in the plasma. Furthermore, the major components responsible for transporting monovalent ions across the luminal membrane of the choroid plexus are Na^+^/K^+^ ATPase, Na^+^/K^+^/2Cl^−^ cotransporter, and channels for the selective secretion of K^+^ and {\text{HCO}}_{3}^{-}] . The net effect of the interaction of these components is the unidirectional influx of Na^+^, Cl^−^, and {\text{HCO}}_{3}^{-}] from the choroid plexus epithelium into the brain ventricle, which forms an osmotic gradient that drives water into the CSF. Na^+^/K^+^ ATPase plays a major role in pumping sodium ions into the CSF and is important for the production of CSF. It also creates electrochemical gradients to drive Cl^−^, and {\text{HCO}}_{3}^{-}] cross the luminal membrane to the CSF [1,20]. In our study, the concentration of Na^+^ in the blood was significantly lower in the DM group (*p* = 0.005). Patients with DM usually have abnormal serum sodium concentrations. This dilutional hyponatremia is caused by an increased serum osmolality due to a high glucose level, which results in shifting of water from cells to extracellular fluid [21]. In the brain, Na^+^/K^+^ ATPase plays a vital role in maintaining the homeostasis of Na^+^ ions in the CSF. In an animal model of induced DM, the expression of Na^+^/K^+^ ATPase in the brain was shown to be associated with age and sex. In older animals, there seemed to be a significant loss of enzyme activity from diabetic insults. In addition, the activity of Na^+^/K^+^ ATPase was significantly lower in the brain of male diabetic animals than in the female group [22]. In our controlled study, though there were more male patients with higher age in DM group, the difference in the concentration of Na^+^ in the CSF between the two groups was not observed. Further studies are needed to investigate the activities of Na^+^/K^+^ ATPase in patients with DM compared to the control. We also found that patients with higher blood glucose levels had higher Na^+^ CSF/blood ratios (Figure 2). This may be attributed to dilutional hyponatremia caused by high blood glucose levels.

Increasing evidence suggests that Ca^2+^ and Mg^2+^ play key roles in the CNS. Ca^2+^ ions control neurotransmitter release, modulate the activity of ion channels, as well as regulate neuronal plasticity and cell death [23,24]. Ca^2+^ and Mg^2+^ in the CSF and serum may also modulate seizure activity [25]. A higher incidence of hypomagnesemia ranging from 13.5 to 47.7% has been reported in patients with type 2 DM [26]. In our study, a lower concentration of Mg^2+^ in the blood was observed in the DM group than in the control group. The contributory factors may be reduced oral intake and gastrointestinal absorption of magnesium due to diabetic autonomic neuropathies and enhanced renal loss. In the CNS, mechanisms influencing the concentration of CSF Mg^2+^ are not fully understood. A previous study showed that prolonged induced hypermagnesemia only leads to a marginal increase in CSF Mg^2+^ in the human brain [27]. Another study revealed that intravenous magnesium infusion did not increase CSF Mg^2+^ concentration in patients with intracranial hypertension [28]. The regulation of CSF Mg^2+^ may be controlled by active ion transport through a specific channel, and Mg^2+^ is maintained at a higher level in the CSF than in the blood. In our study, the concentrations of CSF Mg^2+^ were similar in the DM and control groups, despite the significant difference (*p* = 0.045) in the blood Mg^2+^ concentration between the two groups.

Glucose is the primary metabolic energy source for the mammalian brain. In the CNS, glucose crosses the BBB into the extracellular space of the brain through the glucose transport (GLUT) family, mainly GLUT-1 and GLUT-3. GLUT1 plays an important role in brain glucose uptake and is largely distributed in the BBB and astrocytes [29]. Previous studies undertaken in hyperglycemic animal models have shown that chronic elevated blood glucose downregulates GLUT-1 and GLUT-3 expression in the BBB of the brain [30]. However, another study undertaken in diabetic rats revealed no significant change in the expression of glucose transporters in the BBB [31]. These conflicting results may be due to the different animal models or analytical methods employed. In human adults, the general range of CSF glucose concentration is usually directly proportional to the plasma values and is approximately 50–60% of the plasma levels [32]. In our study, there were no significant differences in the CSF/blood glucose ratio between the control and the DM groups (0.40–0.62 vs 0.44–0.64). A low CSF/plasma glucose ratio (usually <0.4) may be indicative of a CNS infection; either bacterial, or fungal. On the other hand, an elevated CSF glucose concentration usually has no specific pathologic significance and may be merely related to high blood sugar levels [33], as observed in patients with DM in our study.

## 5 Conclusion

Diabetes-induced alterations in blood glucose levels may cause physiological changes. Our results showed that there are some homeostatic disorders of ions, acid-base, and sugar in the blood and CSF of patients with DM. A detailed analysis of BBB function, expression, and activity of glycated proteins that could induce endothelial damage or other solutes such as amino acids, inflammatory mediators, or metabolic products in CSF in DM patients is further indicated.
